# Clinical Findings for Early Human Cases of Influenza A(H7N9) Virus Infection, Shanghai, China

**DOI:** 10.3201/eid1907.130612

**Published:** 2013-07

**Authors:** Shuihua Lu, Yufang Zheng, Tao Li, Yunwen Hu, Xinian Liu, Xiuhong Xi, Qingguo Chen, Qingle Wang, Ye Cao, Yanbing Wang, Lijun Zhou, Douglas Lowrie, Jing Bao

**Affiliations:** Shanghai Public Health Clinical Center, Shanghai, China (S. Lu, Y. Zheng, T. Li, Y. Hu, X. Liu, X. Xi, Q. Chen, Q. Wang, Y. Cao, Y. Wang, L. Zhou, D. Lowrie);; Henry M Jackson Foundation–Division of AIDS, National Institute of Allergy and Infectious Diseases, National Institutes of Health, Bethesda, MD, USA (J. Bao)

**Keywords:** influenza, influenza A(H7N9) virus, subtype H7N9, H7N9, Shanghai, China, case reports, diagnosis, treatment, viruses

## Abstract

A novel strain of influenza A(H7N9) virus has emerged in China and is causing mild to severe clinical symptoms in infected humans. Some case-patients have died. To further knowledge of this virus, we report the characteristics and clinical histories of 4 early case-patients.

Avian influenza A(H7N9) virus normally circulates among birds; however, human infections with this virus were confirmed in China on March 31, 2013 (*1*,*2*). To help identify the best treatment strategies for influenza A(H7N9) virus infection, we summarized the clinical characteristics and outcomes for the first 4 patients who were transferred to Shanghai Public Health Clinical Center (SHPHCC) for treatment of influenza A(H7N9) virus infection. For each case, infection was confirmed by the Shanghai Municipal Centers for Disease Control and Prevention.

## Case Reports

Clinical features of the 4 case-patients are listed in Table 1. All case-patients were 58- to 73-year-old married men, farmers or retirees, and long-term residents of Shanghai (Fengxian, Baoshan, Songjiang, and Pudong districts, respectively). Case-patient 1 had a history of coronary heart disease and hepatic schistosomiasis; case-patient 2 had no history of chronic disease; case-patient 3 had a history of hypertension and gout; and case-patient 4 had a history of hypertension and repetitive cough for >10 years during spring and autumn.

**Table 1:** Clinical characteristics and treatment outcomes for 4 patients with early cases of influenza A(H7N9) virus infection, Shanghai, China*

Characteristic/treatment	Case-patient no.			
	1	2	3	4
Age, y/sex	73/M	65/M	67/M	58/M
Occupation	Farmer	Retiree	Retiree	Retiree
Location (district) in Shanghai	Fengxian	Baoshan	Songjiang	Pudong
Disease history	Coronary heart disease; chronic hepatic schistosomiasis	Hypertension; articular gout; benign prostatic hyperplasia	None	Hypertension
History of poultry exposure	At home	At live poultry markets	At live poultry markets	At live poultry markets
Date of last visit to live poultry market	NA	2013 Mar 29	2013 Mar 28	2013 Mar 19
Date of symptom onset	2013 Mar 31	2013 Apr 1	2013 March 30	2013 Mar 20
Date of infection confirmation	2013 Apr 6	2013 Apr 6	2013 Apr 7	2013 Apr 7
Date admitted to SHPHCC	2013 Apr 6	2013 Apr 6	2013 Apr 7	2013 Apr 7
Clinical symptoms present when admitted SHPHCC	6 d of fever (maximum temperature 39.3°C) and shortness of breath	6 d of fever (maximum temperature 39.3°C), and 2 d of cough	8 d of fever (maximum temperature 39.7°C) and cough	18 d of cough, 10 d of fever (maximum temperature 39.7°C), and 5 d with shortness of breath
Chest radiograph or CT findings	Bilateral GGO	Bilateral GGO	GGO in left lingular lobe and left inferior lobe	Extensive infiltrates, with pleural effusion, in lung (bilateral)
Antiviral drug treatment	Oseltamivir (150 mg/bid) on days 7–12 of illness	Oseltamivir (75 mg/bid) on days 4–17 of illness	Oseltamivir (75 mg/bid) on days 6–21 of illness	Oseltamivir (75 mg/bid) on days 16–23 of illness; oseltamivir (150 mg/bid) on days 17–32 of illness
Antibacterial drug treatment	Moxifloxacin on days 7–12 of illness	Ceftriaxone on days 4–5 of illness; moxifloxacin on days 6–17 of illness	Azithromycin on days 5–9 of illness; cefaclor on days 1–5 of illness; moxifloxacin on days 14–21 of illness	Moxifloxacin on days 18–21 of illness; piperacillin and tazobactam on days 18–21 of illness; meropenem on days 21–34 of illness; linezolid on days 25–32 of illness
Glucocorticoid treatment	Methylprednisolone (40 mg/d) on days 7–12 of illness	No	Methylprednisolone (40 mg/d) on days 5–12 of illness	Methylprednisolone (40 mg/bid) on days 16–37 of illness
Immunoglobulin treatment	Yes, on days 7–12 of illness	Yes, on days 6–12 of illness	Yes, on days 5–8 of illness	Yes, on days 16–37 of illness
ECMO treatment	No	No	No	On day 25 of illness
Oxygen use	Noninvasive ventilation on days 6–12 of illness	Oxygen inhalation through nasal tube on days 4–17 of illness	Oxygen inhalation through nasal tube on days 7-20 of illness	Noninvasive ventilation on days 17–19 of illness
Endotracheal intubation and mechanical ventilation	Yes, on day 12 of illness	No	No	Yes, on days 19–32 of illness
Status as of 2013 Apr 21	Died on day 12 of illness	Recovered, discharged on day 18 after illness onset	Recovered, discharged on day 21 after illness onset	Condition worsened, receiving invasive breath machine and ECMO treatment

*NA, not applicable; SHPHCC, Shanghai Public Health Clinical Center; CT, computed tomography scan; GGO, ground-glass opacity; bid, 2 times a day; ECMO, extracorporeal membrane oxygenation.

Case-patient 1 raised chickens at home. Case-patients 2–4 had no clear history of close contact with poultry; however, each had visited various farmers’ markets that sold live poultry. None of the patients raised pigeons or live in or near a heavily pigeon-infested area.

Before being transferred to SHPHCC on April 6, 2013 (patients 1 and 2) and April 7, 2013 (patients 3 and 4), the 4 patients had been treated in local hospitals; infection with influenza A(H7N9) virus had been confirmed by real-time reverse transcription PCR of nasopharyngeal swab samples before transfer. The case-patients had cough and fever and had been expectorating sputum for ≈6–7 days before admittance to SHPHCC. In addition, all had experienced cold-like symptoms and fatigue before influenza-like symptoms developed. Case-patient 4 had cough and fever for 18 and 10 days, respectively, before being transferred to SHPHCC; his case was the most serious of the 4, and the disease progressed rapidly after he was transferred to SHPHCC.

**Figure 1:**
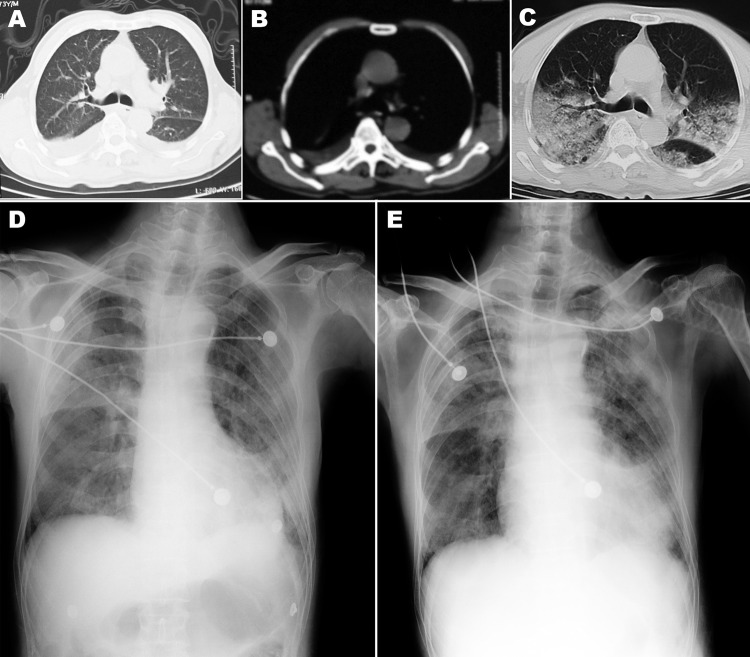
Chest computed tomography (CT) scan and radiograph images of patient (case-patient 1) in a study of 4 persons with early cases of influenza A(H7N9) virus infection, Shanghai, China. Images were taken 1, 5, 7, and 11 days after illness onset. A, B) CT scan images on day 1, showing bilateral pleural effusion but no obvious lesions. C) CT scan image on day 5, showing extensive ground-glass opacity and consolidation. D, E) x-ray images on days 7 and 11, respectively, showing reduced light transmittance on both sides of the lung.

**Figure 2:**
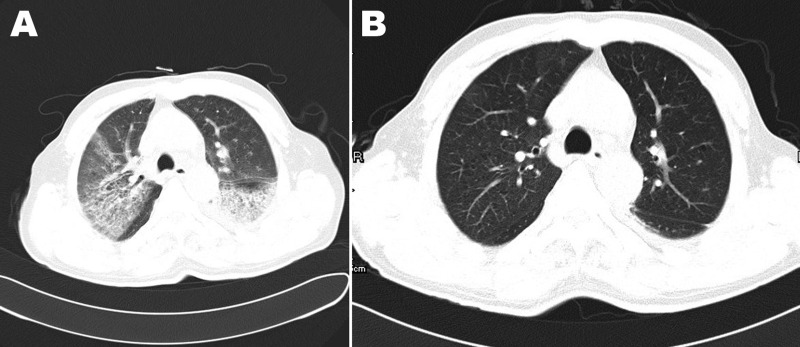
Chest computed tomography scan images of patient (case-patient 2) in a study of 4 persons with early cases of influenza A(H7N9) virus infection, Shanghai, China. A) Image taken 6 days after illness onset shows ground-glass opacity in the left lower and right upper lobes. B) Image taken 16 days after illness onset shows absorption of ground-glass opacity.

Total leukocyte counts for case-patients 1–4 were within or slightly below reference values: 5.50, 5.95, 3.50, and 4.60 × 10^9^/L, respectively (reference value 4.00–10.00 × 10^9^/L). The proportions of neutrophils were normal or slightly high: 79.6%, 62.6%, 72.4%, and 68.0%, respectively (reference value 50.0%–70.0%). Laboratory test results at admission are shown in Table 2. Radiograph findings mainly included ground-glass opacity and consolidation (Figures 1, 2; Technical Appendix Figures 1, 2). Computed tomography (CT) scans and radiograph findings, along with clinical manifestations and laboratory test results, helped establish early diagnoses.

**Table 2:** Laboratory findings at admission for 4 patients with early cases of influenza A(H7N9) virus infection, Shanghai, China

Laboratory variable	Case-patient no.				Reference value
	1	2	3	4	
Leukocyte count, × 10^9^/L	2.95	3.74	2.89	5.38	4.00–10.00
% Neutrophils	80.4	76.7	78.6	94.6	50.0%–70.0%
% Lymphocytes	13.5	18.2	15.4	2.4	20.0%–40.0%
Platelet count, × 10^9^/L	71	82	172	75	85–303
Aspartate aminotransferase, U/L	86	77	45	172	8–40
Lactate dehydrogenase, U/L	886	492	209	906	109–245
Creatine phosphokinase, U/L	170	1,854	170	772	38.00–174
Creatine kinase isoenzyme MB, U/L	18	31	7	22	0–24

To ensure proper treatment/management of the patients, an emergency team was established; the team followed the procedures shown in Technical Appendix Figure 3. All 4 case-patients were administered antimicrobial drugs and the antiviral drug oseltamivir. Case-patient 1 began treatment 6 days after the onset of hypoxia, when large areas of lung inflammation were seen on radiographs. Case-patient 2 was treated 4 days after the onset of fever, when CT scan results revealed inflammation in the left upper lung lobe. Case-patient 3 began treatment 4 days after the onset of cough, sputum, and shortness of breath and after CT scan results revealed inflammation in the left lower lung lobe. Case-patient 4 began treatment 16 days after onset of high fever, dyspnea on exertion, and hypoxemia. Additional details for each patient are included below, and results of viral testing done at admission and 5 days later are shown in Technical Appendix Table 1. Disease characteristics for infections caused by influenza virus subtypes H1N1, H5N1, and H7N9 are shown in Technical Appendix Table 2.

Case-patient 1 was receiving noninvasive ventilator-assisted breathing when he arrived at SHPHCC. His oxygen saturation remained at ≈95%, and he was given continuous intravenous dopamine infusion. He had acute respiratory failure, coronary heart disease (stage 2 heart failure), and renal function insufficiency at admission. On April 11, 11 days after the onset of the symptoms and 2 hours after endotracheal intubation and mechanical ventilation began, he died from respiratory failure.

Case-patient 2 arrived at SHPHCC with a nasal cannula inserted to maintain oxygen saturation at 95%. His general condition improved steadily after commencing antiviral drug treatment, and he was discharged 18 days after illness onset.

Case-patient 3 arrived at SHPHCC with a nasal cannula inserted to maintain oxygen saturation at 95%. He had a history of hypertension and gout. He was treated with oseltamivir, antimicrobial drugs, and steroids to suppress lung inflammation. His condition improved substantially, and he was discharged 21 days after illness onset.

Case-patient 4 arrived at SHPHCC in critical condition: oxygen saturation was 88%, and he had shortness of breath (30–35 breaths/min). He was immediately given noninvasive mechanical ventilation. One day after admission, his condition deteriorated; multiple organ dysfunctions in lung and kidney developed. His condition continued to deteriorate despite active treatment with oseltamivir and antimicrobial drugs. Severe hypoxemia developed. Two days after admission, invasive mechanical ventilation and then extracorporeal membrane oxygenation were implemented. The patient was still in critical condition on April 21, 2013.

## Discussion

Clinical manifestations of disease in the 4 case-patients were consistent with those reported for other persons infected with influenza A(H7N9) virus (*3*). Case-patients 1 and 4 had a more severe disease course than case-patients 2 and 3. All patients sought medical care for unresolved fever, cough, expectoration of sputum, and shortness of breath. The severe cases progressed rapidly: body temperature was mostly sustained >39°C, and breathing was difficult and sometimes accompanied by hemoptysis. A rapid progression of acute respiratory distress syndrome occurred in case-patients 1 and 4, along with mediastinal emphysema, shock, disturbed consciousness, and acute kidney injury. No close contacts of the 4 patients have had signs or symptoms of infection.

The currently available drug treatment for influenza A(H7N9) virus infection is neuraminidase inhibitors (e.g., oseltamivir). Their early use may be recommended (*4*) but is not always achieved. Case-patient 4 only began neuraminidase inhibitors 16 days after the onset of symptoms, by which time he was in a severe condition. Case-patient 1 was treated with oseltamivir 6 days after the onset of symptoms and, despite treatment, died 6 days after admission to SHPHCC. Earlier, higher doses combined with continuous treatment might improve patient outcomes (*5*). On the basis of clinical judgment, we now use 150 mg of oseltamivir twice daily for severe cases, monitoring for toxicity.

The benefits of oseltamivir treatment of influenza A(H7N9) virus infections are debatable; for example, case-patients 2 and 3 remained positive for the virus after 9–11 days of oseltamivir treatment (Technical Appendix Table 1). Thus, it is essential to determine whether the virus has developed resistance to oseltamivir. Ineffectiveness of the oral oseltamivir formulations may also have contributed to treatment failure, especially for case-patients 1 and 4: the drug may not have been well absorbed, especially by patients in severe condition. If available in the future, systemic delivery of oseltamivir may be superior.

Of the 4 patients reported here, only case-patient 1 died shortly after admission to SHPHCC. He is also the only patient who had close contact with chickens. However, it is not clear that this contact contributed to the rapid progression of disease in case-patient 1, especially given the fact that case-patient 4, who is still in critical condition, also had rapid progression of disease. The other patients did not raise birds at home, but they visited live poultry markets.

Prompt and early communication of the clinical features of persons infected with avian influenza A(H7N9) virus is crucial to the development of effective treatment strategies (*6*). Research to understand the transmission pattern and effective control of this virus is urgently needed (*7*–*9*).

Technical Appendix Virus detection in 4 influenza A(H7N9) virus–infected persons in Shanghai, China; variables for infection with influenza A subtypes H1N1, H5N1, and H7N9; computer tomography scan and radiograph findings for case-patients 3 and 4; and outline of procedures followed by emergency team managing/treating case-patients 1–4.
