# Supplementary material for: Clinical Findings for Early Human Cases of Influenza A(H7N9) Virus Infection, Shanghai, China
Source: Emerg Infect Dis. 2013 Jul;19(7):e130612. doi: 10.3201/eid1907.130612 (PMC3713996; doi:10.3201/eid1907.130612)
Supplement: Technical Appendix — Virus detection in 4 influenza A(H7N9) virus–infected persons in Shanghai, China; variables for infection with influenza A subtypes H1N1, H5N1, and H7N9; computer tomography scan and radiograph findings for case-patients 3 and 4; and outline of procedures followed by emergency team managing/treating case-patients 1–4. [file 13-0612-Techapp-s1.pdf]

Article DOI: http://dx.doi.org/10.3201/eid1907.130612

Clinical Findings for Early Human Cases of Influenza A(H7N9) Virus Infection, Shanghai, China

Technical Appendix

Technical Appendix Table 1. Virus detection in 4 patients with influenza A(H7N9) virus infection, Shanghai, China*

	At admission			6–7 Days after admission		
Case-patient	Day of illness	No. day(s) using oseltamivir	Virus detected	Day of illness	No. days using oseltamivir	Virus detected
1	6	1	Yes	12	7	Yes
2	5	2	Yes	12	9	No
3	8	4	Yes	15	11	No
4	18	2	Yes	25	9	Yes

*Patients were admitted to Shanghai Public Health Clinical Center for treatment after confirmation of infection.

Technical Appendix Table 2. Comparison of H1N1, N5N1, and H7N9 in terms of epidemiology, symptoms, chest x-ray images, and disease prognosis

	Influenza virus subtype		
Variable	H1N1	H5N1	H7N9
Epidemiology	Patients as the main source of infection	History of environmental exposure to avian influenza	With or without history of environmental exposure to avian influenza
Symptoms	Cough, expectoration, polypnea, and poor appetite; main signs were moist rales and rough breathing sounds in lungs	Fever, the whole body muscle aches, fatigue, cough, purulent sputum, sometimes with blood or pus, chest pain, diarrhea	Fever, whole body muscle aches, fatigue, cough, purulent sputum, sometimes with blood or pus, chest pain, diarrhea
Chest x-ray images	Pneumonia, with or without pleural effusion	Pulmonary effusion, with or without pleural effusion	Pulmonary effusion, with or without pleural effusion
Disease prognosis	Generally good prognosis	Poor prognosis	Unknown

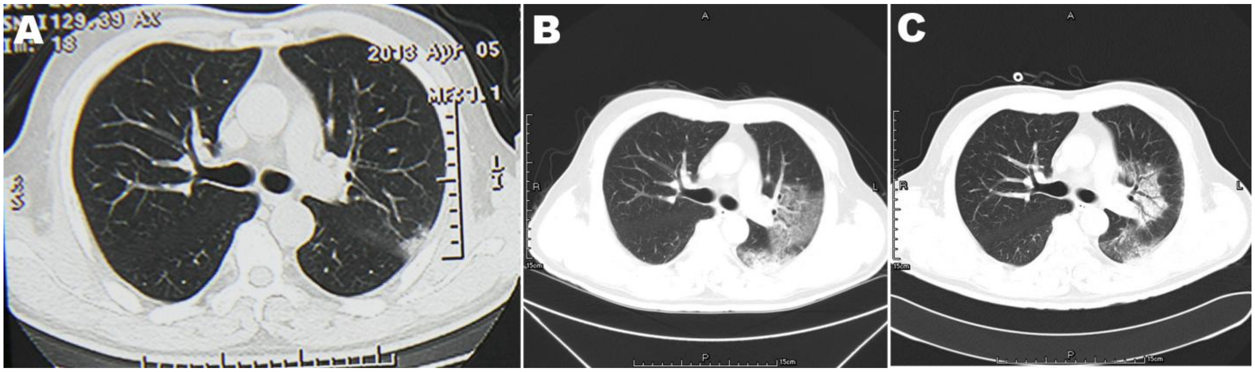

Technical Appendix Figure 1. Chest computed tomography scan images of patient 3, taken on days 6, 8, and 16 after the onset of illness. A) Only a little ground-glass opacity is present in the left upper lobe on

day 6. B) On day 8, the area of ground-glass opacity was enlarged, as seen in the left lingular and inferior lobes. C) Some absorption of the lesions was seen on day 16.

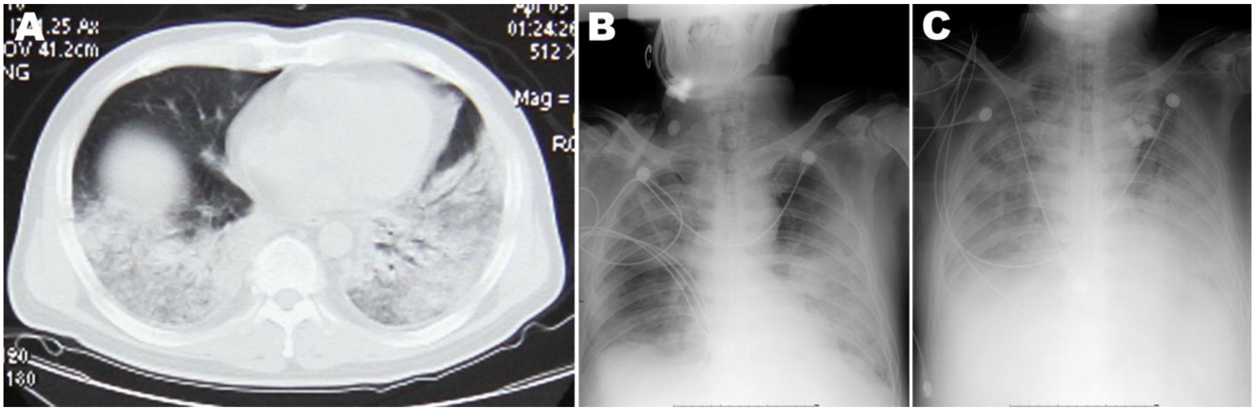

Technical Appendix Figure 2. Chest computed tomography scan and radiograph images of patient 4, taken on days 21, 24, and 28 after the onset of illness. A) The computed tomography scan image shows extensive bilateral lung infiltrates on day 21. B) The radiograph image shows areas of low light transmittance on both sides of the lung on day 24. C) The radiograph image shows larger areas of low light transmittance on day 28.

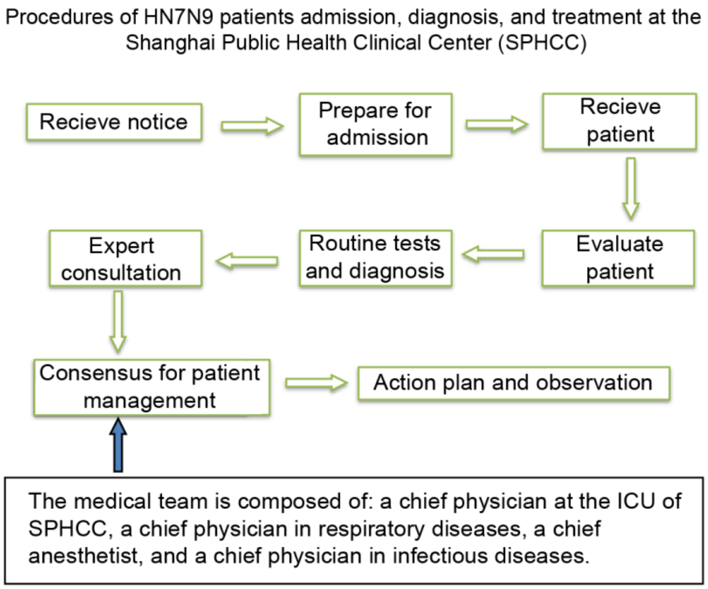

Technical Appendix Figure 3. Procedures followed by an emergency team that was established at Shanghai Public Health Clinical Center (SHPHCC) for managing patients admitted for treatment of

confirmed influenza A (H7N9) virus infection, Shanghai, China. In brief, infection was confirmed and SHPHCC was notified before patients were transferred to the Center. Upon admission to SHPHCC, patients were evaluated thoroughly, and the disease condition was assessed. On the basis of the clinical diagnoses and results of routine laboratory tests, a consensus for managing, monitoring, and treating individual patients was established by a medical team composed of the chief physician in the SHPHCC intensive care unit, a chief physician in respiratory diseases, a chief anesthetist, and a chief physician in infectious diseases.
